# Supplementary material for: Emerging symmetric strain response and weakening nematic fluctuations in strongly hole-doped iron-based superconductors
Source: Nat Commun. 2021 Aug 10;12:4824. doi: 10.1038/s41467-021-25121-5 (PMC8355183; doi:10.1038/s41467-021-25121-5)
Supplement: Supplementary file 1 — Supplementary Information [file 41467_2021_25121_MOESM1_ESM.pdf]

Supplementary Information for: Emerging symmetric strain response and weakening nematic fluctuations in strongly hole-doped iron-based superconductors

P. Wiecki, M. Frachet, A.-A. Haghighirad, T. Wolf, C. Meingast, and R. Heid
Karlsruhe Institute of Technology, Institute for Quantum Materials and Technologies, 76021 Karlsruhe, Germany

A. E. Böhmer
Karlsruhe Institute of Technology, Institute for Quantum Materials and Technologies, 76021 Karlsruhe, Germany and Institut für Experimentalphysik IV, Ruhr-Universität Bochum, 44801 Bochum, Germany
(Dated: July 19, 2021)

SUPPLEMENTARY NOTE 1: SUPPLEMENTARY FIGURES

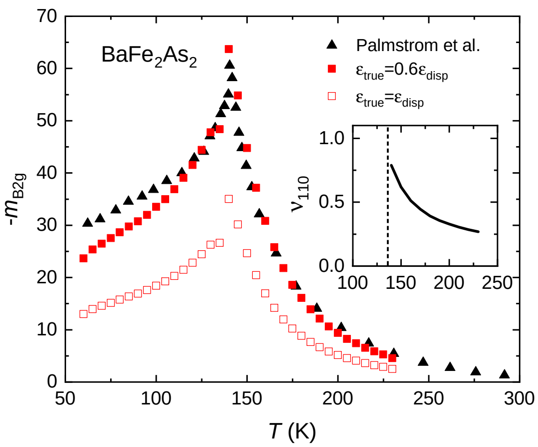

Supplementary Fig. 1. **Comparison of strain cell and piezostack elastoresistance in $BaFe_2As_2$.** B_{2g} elastoresistance coefficient of $BaFe_2As_2$ determined from longitudinal and transverse elastoresistance measurements in our strain cell setup. A comparison with the accepted data of Palmstrom *et al.* [1], obtained by the piezoelectric stack technique, reveals almost perfect agreement when considering that the true strain (ϵ_{true}) in our strain cell is 60% of the nominal strain displayed by the capacitance sensor (ϵ_{disp}). The deviations at low temperature may be due to a different distribution of orthorhombic domains in two measurement setups. The inset shows the Poisson ratio $\nu_{[110]}$ calculated from the experimental temperature-dependent elastic constants c_{11}, c_{12}, c_{33} and c_{44} of $Ba(Fe_{0.963}Co_{0.037})_2As_2$ and c_{66} of $BaFe_2As_2$ extracted from ultrasound measurements [2]. Since experimental data for c_{13} are not available, we take $c_{13} = 20$ GPa, independent of temperature.

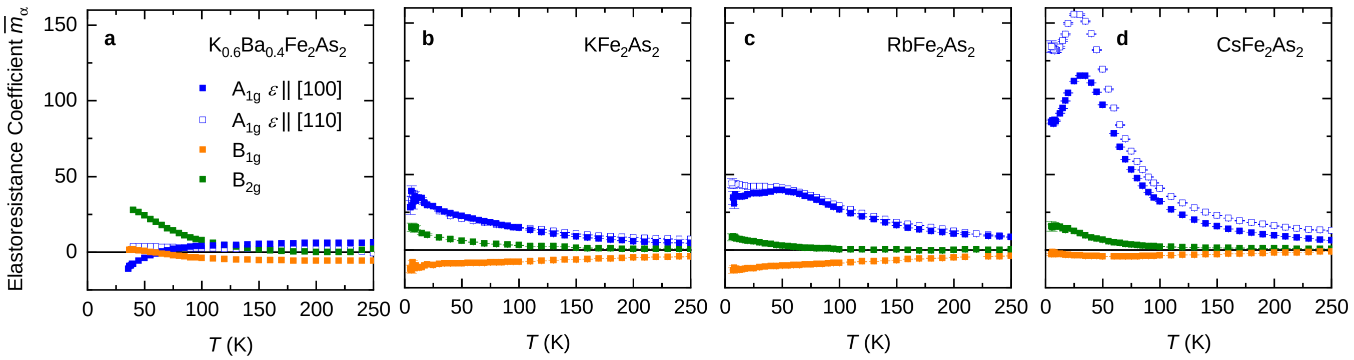

Supplementary Fig. 2. **Symmetry-decomposed elastoresistance coefficients $\bar{m}_\alpha$ grouped by compound.** For the A_{1g} coefficient, the values calculated from both $\epsilon_{xx}\|[100]$ data and $\epsilon_{xx}\|[110]$ data are shown. Differences between $\bar{m}_{A_{1g}}$ calculated from $\epsilon_{xx}\|[100]$ and $\epsilon_{xx}\|[110]$ may be attributed to temperature dependence of the sample Poisson ratio or misalignment of the electrical contacts.

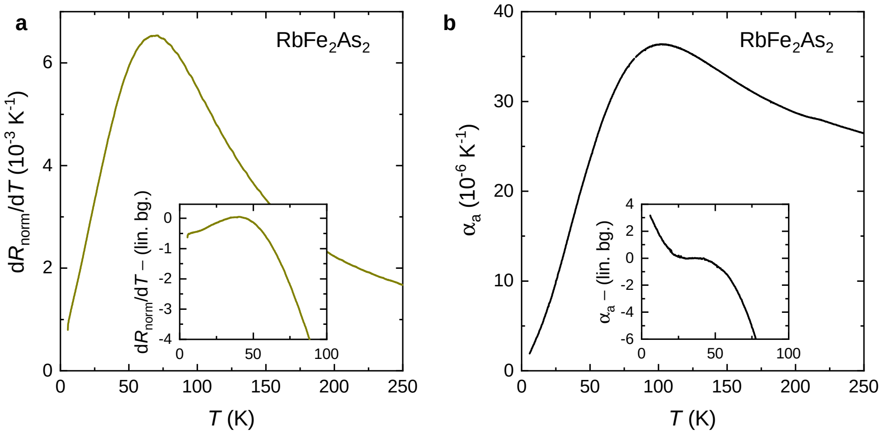

Supplementary Fig. 3. **No evidence for a nematic phase transition in RbFe$_2$As$_2$. a** The temperature derivative of the resistance of RbFe$_2$As$_2$ (normalized at 300 K), showing no sharp anomalies associated with a possible phase transition[3] at the temperature of maximum $\bar{m}_{A_{1g}}$ around 40-50 K. The inset shows the same data, with a linear background subtracted. **b** The uniaxial in-plane thermal expansion coefficient α_a of RbFe$_2$As$_2$, showing no sharp anomalies associated with a possible phase transition around 40-50 K. The inset shows the same data, with a linear background subtracted. Note that the α is expected to be a sensitive probe for pressure sensitive phase transitions.

SUPPLEMENTARY NOTE 2: QUANTITATIVE COMPARISON BETWEEN ELASTORESISTANCE AND THERMODYNAMIC QUANTITIES

The resistance of KFe$_2$As$_2$ can be fit to $R = R_0 + AT^2$ at low temperature. In this Fermi-liquid formula, A is a measure of the effective mass according to $A = c_{\mathrm{KW}}\gamma^2$, where $c_{\mathrm{KW}} = 2 \times 10^{-6}$ $\mu\Omega$ cm(K mol/mJ)^2 is the Kadowaki-Woods constant appropriate for this system[4]. Using this constant, we can compare the strain dependence of the A coefficient (Fig. 3**b** of the main text) with the strain dependence of γ inferred from the thermal expansion.

We consider here the case of KFe$_2$As$_2$ with strain applied along [100] (Fig. 3 of the main text). From thermal expansion in Ref. 4 $d\gamma/d\epsilon_a = 1149.6$ mJ/molK^2 and $d\gamma/d\epsilon_c = 820.44$ mJ/molK^2. The expected change of γ induced by the strain in our experiment is

$$\Delta\gamma = \left[2\frac{d\gamma}{d\epsilon_a} - \nu'_{[100]}\frac{d\gamma}{d\epsilon_c}\right]\Delta\epsilon_a.$$

In this equation we use $\Delta\epsilon_a \equiv \Delta\epsilon_{A_{1g}} = (1 - \nu_{[100]})\Delta\epsilon_{xx}/2$. In our experiment the longitudinal strain amounts to $\Delta\epsilon_{xx} = 0.0042$ (Fig. 3**b** of the main text) giving $\Delta\epsilon_{A_{1g}} = 0.00148$. With the numerical values of $\nu_{[100]}$ and $\nu'_{[100]}$ (see Methods), we obtain $\Delta\gamma = 2.67$ mJ/molK^2 for the expected change of γ in our experiment. Note that this calculation assumes that γ is unaffected by the B_{1g} component of strain.

To calculate $\Delta\gamma$ from resistance measurements, we use $\Delta\gamma = \Delta A/2c_{\mathrm{KW}}\gamma$. Defining $A_{A_{1g}} \equiv (A_{xx} + A_{yy})/2$ we find that the change in $A_{A_{1g}}$ under strain ϵ_{xx} is given by

$$\Delta A_{A_{1g}} = \frac{1}{2}\left[\frac{dA_{xx}}{d\epsilon_{xx}} + \frac{dA_{yy}}{d\epsilon_{xx}}\right]\Delta\epsilon_{xx}.$$

$dA_{yy}/d\epsilon_{xx} = 0.219$ $\mu\Omega$ cm/K^2 is the slope of Fig. 3**b** of the main text. $dA_{xx}/d\epsilon_{xx} = 0.088$ $\mu\Omega$ cm/K^2 is found similarly from the longitudinal elastoresistance data (not shown). To obtain these numbers, we use $\rho(300\text{ K}) = 300$ $\mu\Omega$ cm[5]. Using $\gamma = 100$ mJ/molK^2 for freestanding KFe$_2$As$_2$[6, 7], we then predict $\Delta\gamma = \Delta A_{A_{1g}}/2c_{\mathrm{KW}}\gamma = 1.61$ mJ/molK^2. If we now correct for the fact that the true strain differs from the nominal strain in our strain cell according $\epsilon_{\mathrm{true}} = 0.6\epsilon_{\mathrm{disp}}$ (Fig. 1), we obtain $\Delta\gamma = 1.61/0.6$ mJ/molK^2 $= 2.68$ mJ/molK^2 from the elastoresistance, in agreement with the value $\Delta\gamma = 2.67$ mJ/molK^2 expected from the thermodynamic data calculated above.

A similar comparison for RbFe$_2$As$_2$ and CsFe$_2$As$_2$ is not possible because the low-temperature resistance is well fit by $R = R_0 + AT^n$ with $n \neq 2$, due to non-Fermi liquid behavior. The simple interpretation of the A coefficient as effective mass does not hold in this case.

We can also compare the strain dependence of T_c with values inferred from thermodynamic measurements via Ehrenfest relations[4]. For the case of uniaxial stress, we have that

$$\frac{\Delta T_c}{\Delta\epsilon_a} = \frac{\partial T_c}{\partial\epsilon_a} - \nu\frac{\partial T_c}{\partial\epsilon_a} - \nu'\frac{\partial T_c}{\partial\epsilon_c}. \qquad (1)$$

For the case of KFe_2As_2, we have $\partial T_c/\partial \epsilon_a = 162.12$ K and $\partial T_c/\partial \epsilon_c = 53.24$ K from thermodynamic data in Ref. [4]. With strain applied along [100], using $\nu_{[100]}$ and $\nu'_{[100]}$ (see Methods), we obtain $\Delta T_c/\Delta \epsilon_a = 82.18$ K. Our directly measured value is $\Delta T_c/\Delta \epsilon_a = 103.77 \pm 11.61$ K. Correcting for $\epsilon_{\rm true} = 0.6\epsilon_{\rm disp}$ (Fig. 1), we obtain $\Delta T_c/\Delta \epsilon_a = 62.3 \pm 7.0$ K in reasonable agreement with the thermodynamic value extracted from Ref. [4] ($\Delta T_c/\Delta \epsilon_a = 82.18$ K).

SUPPLEMENTARY NOTE 3: QUALITATIVE COMPARISON BETWEEN ELASTORESISTANCE AND THERMAL EXPANSION

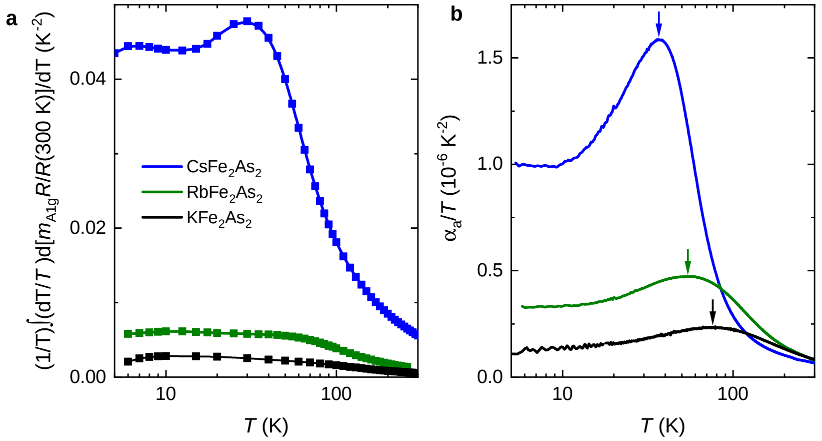

Supplementary Fig. 4. **Qualitative comparison of the temperature dependence of A_{1g} elastoresistance and thermal expansion α_a/T.** The comparison is based on the Fisher-Langer relation (see text). The arrows in panel **b** represent the maxima of the curves, which can be identified as the coherence-incoherence crossover temperature, T^*[7].

The coherence-incoherence crossover in these materials is clearly seen as a maximum in the thermal expansion coefficient divided by temperature α/T[6, 7], which is a measure of the pressure dependence of the entropy. Since the resistance is known to be sensitive to the electronic entropy in these materials[8, 9], the strain derivative of resistance is expected to be related to α/T. In particular, α_a/T measures the change in entropy in response to in-plane symmetric stress, and therefore should probe similar physics as the A_{1g} elastoresistance coefficient $m_{A_{1g}}$. To compare the temperature dependence of $m_{A_{1g}}$ with the temperature dependence of α_a/T, we start from the Fisher-Langer relation for the resivitivy ρ in quantum critical magnets[10–13]

$$\frac{\partial \rho}{\partial T} \propto C_{\rm mag}, \tag{2}$$

where $C_{\rm mag}$ is the magnetic specific heat capacity. Using resistance R in place of ρ, it follows that

$$\frac{1}{T}\frac{\partial R}{\partial T} \propto \frac{\partial S_{\rm mag}}{\partial T}. \tag{3}$$

Taking the pressure derivative of both sides while treating R and $S_{\rm mag}$ as multivariable functions of T and in-plane pressure p, we find that

$$\frac{1}{T}\frac{\partial}{\partial T}\left(\frac{\partial R}{\partial p}\right) \propto \frac{\partial}{\partial T}\left(\frac{\partial S_{\rm mag}}{\partial p}\right). \tag{4}$$

The thermal expansion coefficient α is defined as $\alpha = (-1/V_m)\partial S/\partial p$, where S is the molar entropy and V_m is the molar volume. Here, we make the assumption that $\partial S/\partial p$ is dominated by the magnetic contribution in quantum critical magnets. Therefore, we have

$$\frac{1}{T}\frac{\partial}{\partial T}\left(\frac{\partial R}{\partial p}\right) \propto -\frac{d\alpha}{dT}. \tag{5}$$

One can integrate this equation to obtain

$$\frac{\alpha}{T} \propto -\frac{1}{T}\int \left[\frac{1}{T}\frac{\partial}{\partial T}\left(\frac{\partial R}{\partial p}\right)\right] dT. \tag{6}$$

Here, the integration constant is chosen that $\alpha \to 0$ as $T \to 0$, in accordance with the third law of thermodynamics. To make contact with elastoresistance, we use $(\partial R/\partial \epsilon)_{A_{1g}} = -c_{A_{1g}}(\partial R/\partial p)_{A_{1g}}$, where the minus sign arises because pressure is defined as positive for compression, while strain is defined as positive for tension. We then obtain

$$\frac{\alpha_a}{T} \propto \frac{c_{A_{1g}}}{T} \int \left[\frac{1}{T} \frac{\partial}{\partial T} \left(\frac{\partial R}{\partial \epsilon} \right)_{A_{1g}} \right] dT, \tag{7}$$

where $c_{A_{1g}}$ is the elastic constant for symmetric in-plane stress, which we take to be temperature independent for simplicity. The elastoresistance coefficient is $m_{A_{1g}} = (1/R)(\partial R/\partial \epsilon)_{A_{1g}}$, so that $m_{A_{1g}} R = (\partial R/\partial \epsilon)_{A_{1g}}$. In Fig. 4, we make this comparison and find qualitative agreement between thermal expansion and elastoresistance. However, for $RbFe_2As_2$ and KFe_2As_2 the coherence-incoherence crossover is not as clear in elastoresistance. This may relate to the fact the Fisher-Langer relation applies only in a quantum critical region. $CsFe_2As_2$ is known to be the most critical of these materials[4, 14]. Discrepancies may also be due to a temperature dependence of the elastic constant $c_{A_{1g}}$.

[1] J. C. Palmstrom, A. T. Hristov, S. A. Kivelson, J.-H. Chu, and I. R. Fisher, Phys. Rev. B **96**, 205133 (2017).

[2] M. Yoshizawa and S. Simayi, Modern Physics Letters B **26**, 1230011 (2012), https://doi.org/10.1142/S0217984912300116.

[3] K. Ishida, M. Tsujii, S. Hosoi, Y. Mizukami, S. Ishida, A. Iyo, H. Eisaki, T. Wolf, K. Grube, H. v. Löhneysen, R. M. Fernandes, and T. Shibauchi, Proceedings of the National Academy of Sciences **117**, 6424 (2020), https://www.pnas.org/content/117/12/6424.full.pdf.

[4] F. Eilers, K. Grube, D. A. Zocco, T. Wolf, M. Merz, P. Schweiss, R. Heid, R. Eder, R. Yu, J.-X. Zhu, Q. Si, T. Shibauchi, and H. v. Löhneysen, Physical Review Letters **116**, 237003 (2016).

[5] V. Taufour, N. Foroozani, M. A. Tanatar, J. Lim, U. Kaluarachchi, S. K. Kim, Y. Liu, T. A. Lograsso, V. G. Kogan, R. Prozorov, S. L. Bud'ko, J. S. Schilling, and P. C. Canfield, Physical Review B **89**, 220509 (2014).

[6] F. Hardy, A. E. Böhmer, D. Aoki, P. Burger, T. Wolf, P. Schweiss, R. Heid, P. Adelmann, Y. X. Yao, G. Kotliar, J. Schmalian, and C. Meingast, Physical Review Letters **111**, 027002 (2013).

[7] F. Hardy, A. E. Böhmer, L. de' Medici, M. Capone, G. Giovannetti, R. Eder, L. Wang, M. He, T. Wolf, P. Schweiss, R. Heid, A. Herbig, P. Adelmann, R. A. Fisher, and C. Meingast, Physical Review B **94**, 205113 (2016).

[8] P. Wiecki, A.-A. Haghighirad, F. Weber, M. Merz, R. Heid, and A. E. Böhmer, Phys. Rev. Lett. **125**, 187001 (2020).

[9] Y. Wu, D. Zhao, A. Wang, N. Wang, Z. Xiang, X. Luo, T. Wu, and X. Chen, Physical Review Letters **116**, 147001 (2016).

[10] M. E. Fisher and J. S. Langer, Phys. Rev. Lett. **20**, 665 (1968).

[11] N. Sakamoto, T. Kyômen, S. Tsubouchi, and M. Itoh, Phys. Rev. B **69**, 092401 (2004).

[12] S. M. Stishov, A. E. Petrova, S. Khasanov, G. K. Panova, A. A. Shikov, J. C. Lashley, D. Wu, and T. A. Lograsso, Phys. Rev. B **76**, 052405 (2007).

[13] C. Meingast, Q. Zhang, T. Wolf, F. Hardy, K. Grube, W. Knafo, P. Adelmann, P. Schweiss, and H. v. Löhneysen, in *NATO Science for Peace and Security Series B: Physics and Biophysics* (Springer Netherlands, 2009) pp. 261–266.

[14] Z. T. Zhang, D. Dmytriieva, S. Molatta, J. Wosnitza, S. Khim, S. Gass, A. U. B. Wolter, S. Wurmehl, H.-J. Grafe, and H. Kühne, Physical Review B **97**, 115110 (2018).
